# What makes each of us unique? The nine-banded armadillo as a model to study individuality

**DOI:** 10.3389/fmamm.2024.1450655

**Published:** 2024-09-02

**Authors:** Delva P. Leao, Alvaro Duque, Marcelo O. Dietrich

**Affiliations:** 1 Laboratory of Physiology of Behavior, Department of Comparative Medicine, School of Medicine, Yale University, New Haven, CT, United States; 2 Graduate Program in Biological Sciences-Biochemistry, Universidade Federal do Rio Grande do Sul, Porto Alegre, RS, Brazil; 3 MacBrain Resource Center, School of Medicine, Yale University, New Haven, CT, United States; 4 Department of Neuroscience, School of Medicine, Yale University, New Haven, CT, United States

**Keywords:** *Dasypus novemcinctus*, brain development, mammalian brain development, stochastic developmental variation, comparative & evolutionary neuroscience

## Abstract

The human brain is the foundation of our identity as a species and as individuals. It is where our unique sensations, emotions, and thoughts arise. The same way no two individuals are alike, no two brains are identical. Understanding the expression of inter-individual differences in brain and behavior and their underlying biological mechanisms can profoundly influence neuroscience and the science of individuality. Here, we argue that the nine-banded armadillo is a unique organism for the study of how inter-individual differences are expressed in the mammalian brain. Our argument is based on the fascinating reproductive biology of armadillos, the only known mammals that always generate offspring that are genetic clones, and on how this characteristic can help understand the complex interplay between genetic, environmental, and stochastic factors in the biology of individuality. We will first review the sources of variance in brain-related traits and behavior, then the biology of armadillos, and finally how they can aid in understanding the origins of variance in brain structure and function. Finally, we will provide an overview of the type of studies that can be performed using armadillos and how these studies can advance the science of individuality.

## The contribution of genetics and environment to trait variance

Every individual possesses a unique genetic makeup, inheriting an equal share of genetic material from both parents. The diversity found within human genetics is vast; for example, a study involving the genomes of 2,504 individuals from 26 populations uncovered over 88 million common variants (single nucleotide polymorphisms, short insertions/deletions, and structural variants), highlighting the extensive range of genetic variation among humans (Genomes Project et al., 2015). Such genetic differences play a crucial role in the variances observed in brain structure and function, as well as in individual behavioral and psychological tendencies. However, as we will describe in more detail below, genetic differences cannot account for all the variance observed in phenotypes.

One compelling piece of evidence for the significant impact of genetics on trait variance comes from inbreeding studies. For millennia, humans have practiced inbreeding among animals to select for desired traits. This is evident in the domestication of wolves, leading to the diverse range of modern dog breeds, each with specific characteristics (Lindblad-Toh et al., 2005; Wang et al., 2016). An additional notable example is the fox domestication project in Russia (Belyaev, 1969; Belyaev and The Wilhelmine, 1979; Trut, 1999; Trut et al., 2004). Wild foxes with propensity for human interaction were selected for breeding with the goal of understanding how many generations it would take to tame them. Within just a decade, the selected foxes began to exhibit dog-like behaviors and physical traits, such as increased docility, curly tails, and floppy ears, indicating rapid domestication. Despite critiques suggesting that some conclusions of the fox experiment may have been overstated (Lord et al., 2020), these changes in behavior underscore the principle that for selection to influence a trait, the trait must have a genetic basis. Inbreeding studies, widely utilized in animal research, powerfully demonstrate the influence of genetics on behavioral phenotypes.

A second source of evidence stems from twin and adoption studies (Box 1). Twin studies estimate that the heritability for brain phenotypes varies widely depending on the phenotype analyzed, ranging from 10 to 80% (Jahanshad et al., 2010; Winkler et al., 2010; Blokland et al., 2012). For example, differences in brain asymmetry are region specific. While genetic factors account for 37% of the variance in asymmetry in the anterior thalamic radiation, they only account for 20% of the variation in asymmetry in the forceps major (Lord et al., 2020). A meta-analysis of neuroimaging phenotypes in twins also demonstrates the extent of heritability in brain phenotypes (Winkler et al., 2010). For global volumes, total brain volume exhibits 82% heritability, while cerebral grey matter shows 67% heritability. For cortical thickness, the left frontal lobe shows heritability of 76%, contrasting with the right medial frontal gyrus, which has a heritability of 36% (Winkler et al., 2010). In support of the findings in twin studies, large-scale studies of unrelated individuals demonstrate that the cumulative effect of genome-wide genetic variation can explain only a portion of the variance in brain structure, such as 44% in brain volume and 54% in intracranial volume (Toro et al., 2015). An alternative approach utilizing extended pedigrees to investigate the role of genetics on brain phenotypes confirms the findings consistently observed in twin studies (Jahanshad et al., 2010). Together, these results reinforce the idea that heritability for brain phenotypes varies considerably according to trait and region analyzed and genetic differences cannot account for all the variance observed in the brain.

In line with these findings, adoption studies demonstrate that shared environmental factors account for a comparatively minor portion of trait variance. For example, adoptive siblings, despite being raised in the same family, do not show more resemblance to each other than children raised in different households. Likewise, monozygotic twins raised apart exhibit a striking similarity to those raised together (Bouchard et al., 1990). Collectively, these findings suggest that while genetic variation and environmental factors are key determinants of phenotypes, their sum and interaction cannot fully explain phenotypic variance, indicating the existence of other sources of variance. This is of particular relevance for the brain given its complex development and the capacity to influence many behavioral phenotypes.

## The contribution of a third component to trait variance

To explain what accounts for the remaining variance, it is helpful to revise the pioneering work of Klaus Gärtner (Gartner, 1990). In over two decades of experiments, Gärtner used similar methods from twin studies to investigate the variance in several quantitative traits, such as body weight and organ weight, in laboratory rats, mice, and cattle. For example, to create monozygotic twins, Gärtner split 8-cell stage zygotes in two halves and transferred each half to a different surrogate mother. Gärtner then compared phenotypic differences between these genetically identical animals. In these experiments, 70–80% of the variance in body weight could not be explained by genetic and shared environment factors, arguing for the existence of a third component that explains the variance (Gartner, 1990) (See Table 1 for terminology used in this manuscript, which is largely inspired by the work and writing of Gunter Vogt; for a more in-depth exploration of these concepts, we recommend reading Vogt, 2015, 2020). This third component represents the effects of stochasticity over the course of development. Every biological process, across all levels of organization from gene expression to complex patterns of behavior, show a degree of stochasticity (Kaern et al., 2005; Raser and O’Shea, 2005; Oates, 2011). In other words, these processes are not perfectly deterministic, they exhibit a degree of randomness or noise, and are therefore probabilistic in nature (Honegger and de Bivort, 2018).

To measure the effects of stochasticity, it is important to study clonal organisms—populations of individuals that are genetically identical. Pioneering research from the 1970s by Goodman and colleagues on grasshoppers best illustrates this concept (for a review of this body of work, refer to Goodman, 1978; Goodman et al., 1979). Crucial to these studies were two unique aspects of grasshopper biology: the distinct anatomy of specific neuronal groups and the capability for parthenogenesis, which facilitates the creation of genetically identical offspring. These characteristics made grasshoppers a suitable model for studying the genetic and non-genetic (see Table 1) influences on neuronal variability.

In addition to their two compound eyes, grasshoppers possess three smaller eyes known as ocelli, each connected to receptor cells that synapse with ocellar interneurons. This neuronal network comprises two distinct types of interneurons: a group of 17 large cells and a cluster of approximately 61 smaller cells. The ability to stain these large interneurons consistently and reproducibly and their arborizations with cobalt dye led to a series of studies into the number, placement, and morphology of these neurons. These investigations revealed, for example, a strong genetic basis for the number of large interneurons. Certain isogenic lines exhibited consistent deviations in neuron counts, either an increase or decrease, which was specific to the genotype and not observed across different isogenic lines. However, the spatial distribution of these neurons within the ganglia appeared to be influenced by non-genetic factors, showing variability of several hundred micrometers. Despite this variance in location, the axonal pathways to their target regions exhibited minimal variability, approximately 20 μm. It was also observed that the presence (or not) of synapses with target cells was genetically controlled, but the number of such synapses was not.

From these studies in grasshoppers, it is clear that even in genetically identical individuals raised in nearly identical environments, a large degree of inter-individual differences persists for brain-related phenotypes. These individual differences are the product of the third component, which represents the cumulative effects of stochastic processes that occur during development—or stochastic developmental variation (SDV) (Table 1). SDV is therefore a fundamental property of biology and influences all phenotypes (Hiesinger and Hassan, 2018).

Additional studies in several other species of highly inbred, isogenic, or clonal models aid in demonstrating the role of SDV in creating behavior variability. In inbred lines of fruit flies, for example, systematic assessment of spontaneous locomotor behavior revealed that some lines have consistently elevated levels of intragenotypic variability amongst individuals (Ayroles et al., 2015). Similarly, clonal pea aphids (*Acyrthosiphon pisum*) exhibit consistent behavioral differences in escape response upon predatory attacks (Schuett et al., 2011) and worms display consistent, non-genetic, biases in spontaneous foraging behaviors (Stern et al., 2017). A remarkable study in the naturally clonal fish amazon molly (*Poecilia formosa*) shows that individual variation is present on the first day after hatching. These differences gradually strengthen over a 70-day observation period and are sufficient to differentiate adult individuals (Laskowski et al., 2022; but see also Bierbach et al., 2017). These studies collectively demonstrate that development imparts variation in brain and behavior in genetically identical individuals raised in nearly identical environmental conditions.

## Stochastic developmental variation causes individual differences in brain and behavior

More recently, Hassam and colleagues provided the first causal demonstration of a link between SDV in brain wiring and individual differences in behavior (Linneweber et al., 2020). To provide this demonstration, these scientists studied the fruit fly (*drosophila melanogaster*) visual system. Here, interneurons called dorsal cluster neurons (DCN) exhibit wiring variability between individuals and within the same individual, between the left and right hemispheres of the brain (Zheng et al., 2006).

These DCNs innervate one of two target areas, the medulla or the lobula (Hassan et al., 2000). The decision of each neuron to innervate the medulla or lobula results from a stochastic process (Langen et al., 2013) (see Hiesinger and Hassan, 2018 for a more in-depth review of individual variation in brain wiring). Linneweber and colleagues demonstrated that the number of DCNs varied from 22 to 68, with a range of 11 to 55 targeting the lobula and 6 to 23 targeting the medulla. Using a visual behavioral assay to analyze object orientation responses in flies, the degree of asymmetry in the left-right wiring of the medulla by DCNs determined the behavioral performance of individual flies in this visual guided test. The behavioral performance of individuals was stable over time but showed variability among isogenic individuals. Using an approach similar to what we discussed above for animal domestication, the behavioral individuality of flies in this test was shown to be nonheritable, as inbreeding of flies selected for extremes of the behavior did not result in bias to the phenotype in the offspring. The offspring displayed the full range of behavior variability in the population at every generation, further demonstrating that the behavior is caused by stochastic processes. Thus, this body of work on the fruit fly visual system presents compelling evidence for the striking effect that SDV can have on brain wiring and behavior.

SDV posits that while the process of development is precise, adaptable, and robust, it is also variable. This inherent variability does not negate the precise nature of development or its ability to ensure reliable outcomes. This explains why genetically identical individuals display varying phenotypes. This is as true in worms as it is in humans. Although remarkable progress has been made in understanding the principles and mechanisms of SDV in model species, particularly invertebrates, the translation of these mechanistic insights to the mammalian brain remains mostly a matter of speculation. Indeed, the degree to which SDV impacts mammalian brain development and individual variation in brain and behavior remains very challenging to study due to scarcity of models in which genetic and environmental variation can be controlled simultaneously and separately. Luckily, as Nobel Laureate August Krogh famously stated almost a hundred years ago, “For a large number of problems there will be some animal of choice or a few such animals on which it can be most conveniently studied.”

In the mammalian tree of life, armadillos are the only known mammals that always give birth to genetically identical offspring (Loughry and McDonough, 2013). The armadillo stands as a model capable of filling a significant void in the science of individuality, more closely recapitulating aspects of human physiology than invertebrate models do, thereby offering an opportunity to study inter-individual differences in mammalian brain and behavior that arise from non-heritable origins.

## Armadillos always generate genetically identical offspring

**FIGURE 1:**
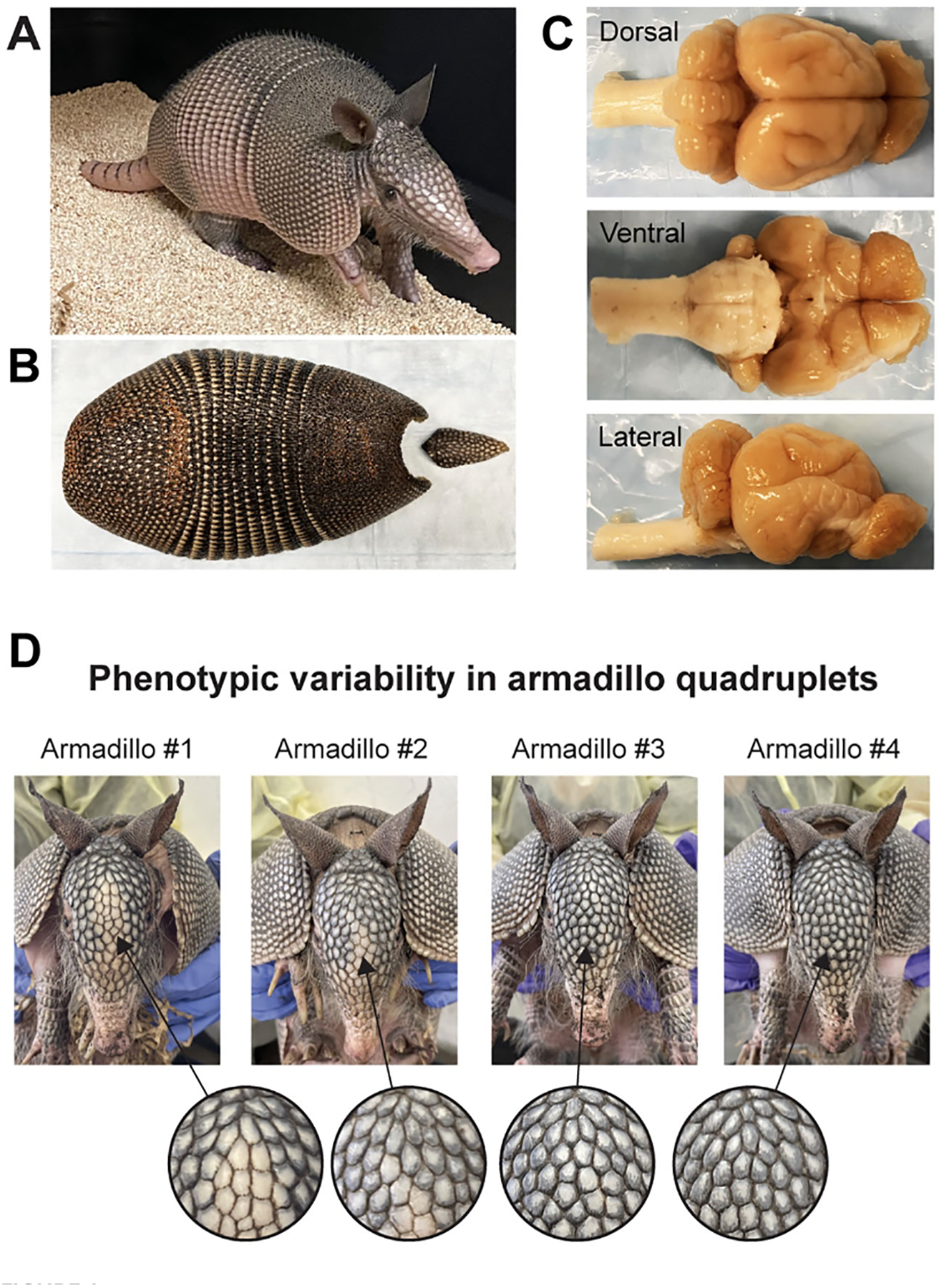
The nine-banded armadillo (*Dasypus novemcinctus*). **(A)** A juvenile nine-banded armadillo raised in our laboratory at Yale University. **(B)** The corresponding carapace showing the nine bands that give the name to this species. **(C)** Dorsal, ventral, and lateral views of this individual’s brain. **(D)** Phenotypic variability in armadillo quadruplets: This set of quadruplets was born in captivity and raised in our laboratory. Note the differences in scale patterning and coloration of the face of individual armadillos. Differences in scale patterning can be observed from early embryonic development and are the result of stochastic developmental processes. Similar processes occur in the development of the nervous system, generating variability in genetically identical individuals.

The nine-banded armadillo (*Dasypus novemcinctus*; hereafter, armadillo) is a distinctive mammal with bony armor covering its body (Figure 1) (McBee and Baker, 1982; Loughry and McDonough, 2013). Armadillos are part of the ancient lineage of placental mammals Xenarthra, with the first fossil records dating back approximately 65 million years. Found in North, Central, and South America, armadillos thrive in forests, grasslands, and near rivers. Known to be solitary foragers and primarily nocturnal with a sharpened sense of smell, their appearance and peculiar reproductive biology has attracted the minds of many scientists yielding an extensive bibliography that spans behavior, physiology, and genetics (Loughry and McDonough, 2013). Pioneer work by Eleanor Storrs in the early 1970s showed that, together with humans, nine-banded armadillos are the only known natural hosts to the pathogen *Mycobacterium leprae*, which causes leprosy. The discovery, resting in Storrs observation of armadillos’ adaptive low basal body temperature, was described as “linking armadillos and man” (Storrs, 1971; Storrs et al., 1974; Patterson, 2018).

Armadillos are part of the genus *Dasypus*, the only group of mammals that presents mandatory polyembryony, a unique reproductive strategy that occurs when a fertilized egg *always* splits into multiples, naturally originating genetically identical individuals (Loughry et al., 1998). In the case of the nine-banded armadillo, the twinning occurs right after the implantation of the blastocyst into the uterine wall. At this moment, the embryo expands and begins to form two distinct sets of embryonic tissues. Subsequently, two more are created at right angles to the first two (Enders, 2002; Loughry and McDonough, 2013). Because all four embryos are derived from a single fertilized egg, they are genetically identical to one another (Prodohl et al., 1996). Initially, all four are contained within a single amnion, but this changes as the amnion collapses into separate amnions for each embryonic disk. Albeit with distinct blood supplies (i.e., umbilical cords), all four embryos share the same placenta. Like the human placenta, the armadillo placenta has only one layer of trophoblast between the maternal blood space and fetal vessels (villous haemomonochorial) (Enders, 1965; Carter, 2021) and hence communication from the maternal to the fetal environment is more homogeneous than in other animals. In other words, the environment is consistent across identical quadruplets during normal embryonic and fetal development.

These reproductive characteristics underscore the armadillo’s importance as a unique mammal for understanding how individual differences arise during development (Ballouz et al., 2023). Here, we propose that the comparative study of clonal armadillos can provide fundamental insights in how inter-individual differences are expressed in the mammalian brain despite nearly identical genetic and shared environment during prenatal development.

## Studying the brain of armadillos at multiple scales

The fact that armadillos always give birth to quadruplets that share the same embryonic environment within one placenta (McBee and Baker, 1982; Loughry and McDonough, 2013) provides a natural experimental paradigm in which genetic and shared environmental factors are controlled. Hence, by measuring any given trait within the set of quadruplets, it is possible to estimate the contribution of the non-genetic factors, shared environment and SDV, in phenotypes (Vogt, 2015, 2020; refer to the pioneer work of Elanor Storrs for measurements of trait variability in armadillo quadruplets: Burchfield, 1967; Storrs and Williams, 1968) (Figure 1). In addition, by comparing the variance among different sets of quadruplets, which are genetically different and don’t share the same environment, it is possible to estimate the contribution of genetic and shared environmental factors (Bagatto et al., 2000; Loughry and McDonough, 2002). Finally, because each set of quadruplets can be split in at least two groups of two, it is possible to perform interventions after birth, such as adoption by surrogate mothers or artificial rearing. These interventions allow to estimate the effects of post-natal environmental factors on individual differences in brain and behavioral phenotypes.

While human twin and adoption studies can shed light on estimated contributions of genetics and shared environment to certain traits, these studies are limited to some phenotypic characteristics usually obtained and measured by brain imaging, blood biomarkers, and behavioral questionnaires. Thus, the use of animal models becomes imperative to study the sources of variance in the brain at the molecular, cellular, and system levels, and to further study the underlying mechanisms. Here, we will highlight examples of problems in the incipient science of individuality that can be advanced by studying brain development of the armadillo.

Example 1: Individual differences in somatic mutations during brain development

Beyond inherited germline mutations, postmitotic cells also accrue somatic mutations throughout an individual’s life, starting from the initial stages of postzygotic cell division. For example, at 20 weeks of gestation, neural progenitors already have accumulated between 200 to 400 somatic mutations (Bae et al., 2018). These mutations tend to amass more rapidly during early embryonic development, with an estimated three new mutations per cell division during the first three divisions, before this rate declines to approximately one new mutation per division thereafter (Rodin et al., 2021; Bae et al., 2022; Lai et al., 2022). Intriguingly, somatic mutations are not exclusive to dividing cells; even non-dividing cells, such as neurons, will accrue about 2,000 somatic mutations over their lifetime, with an average rate of roughly two new mutations per neuron per year (Lodato et al., 2015, 2018).

Somatic mutations, while contributing to variability during typical brain development, may also have deleterious effects. For instance, individuals with autism spectrum disorder often carry a higher number of somatic mutations within neural enhancer sequences, suggesting a link between these mutations and the disorder (Rodin et al., 2021). Recent findings have also uncovered somatic mutations in genes linked to cortical development malformations, such as focal cortical dysplasia, with mutation frequencies varying widely from 1.7% to 44% (Lai et al., 2022). Moreover, in some humans, an elevated number of somatic mutations can be observed, leading to a state of hypermutability. This phenomenon has been observed not only in neurotypical individuals but also in those with neurological and psychiatric conditions, including Tourette syndrome, autism spectrum disorder, and schizophrenia (Bae et al., 2022). From these studies, it is clear that somatic mutations accumulate in unique patterns throughout an individual’s lifespan, with each person harboring a distinctive set of mutations.

However, if the same individual were to develop multiple clones of themselves, would similar or distinct profile of somatic mosaicism emerge? Are hypermutable individuals and individuals with mutations in disease-associated genes the result of random events during brain development? We propose that examining somatic mutations in the brains of genetically identical armadillos offers a promising avenue to answer these questions and shed light into the degree to which somatic mosaicism in the brain stems from pure stochastic processes.

Example 2: Individual differences in cellular and circuit organization in the mammalian brain

**FIGURE 2:**
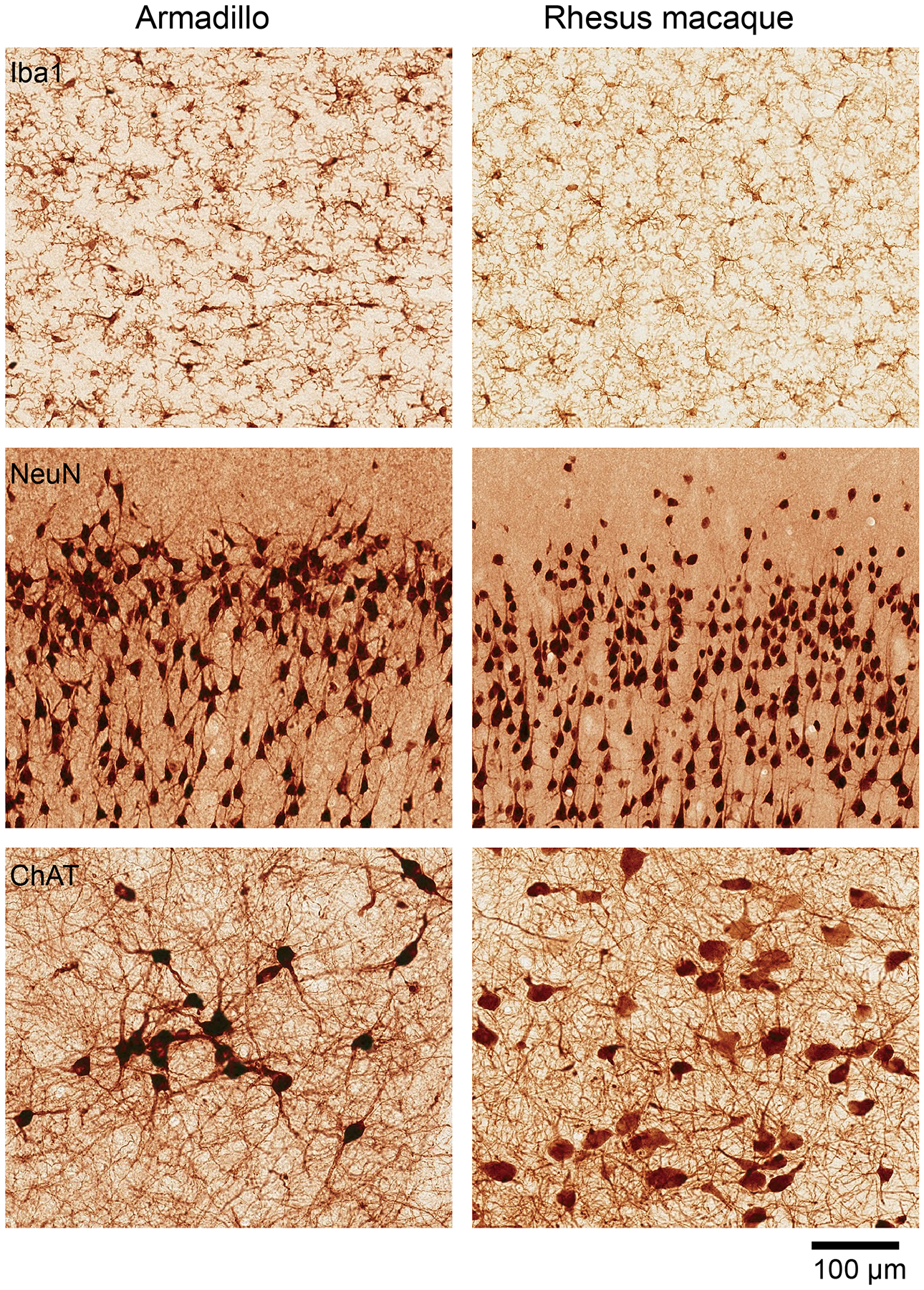
Cellular organization of the armadillo brain. Similar cellular organization between the armadillo and the rhesus macaque brains. Immunohistochemistry based comparison of microglia (stained with Iba1) in the cingulate cortex, pyramidal neurons in the motor cortex (stained with NeuN), and cholinergic neurons in the basal forebrain (stained with choline acetyl transferase). Visualization done by precipitation of DAB. The rhesus macaque used for comparison is a 75-days old female (B66), sections publicly available in Collection 6 of the MacBrain Resource Center (MBRC): https://macbraingallery.yale.edu/collection6/. Scale bar applies to all panels.

The nervous system is characterized by a remarkable consistency in neuronal types and brain connectivity. This consistency underlines the fundamental principles governing neural organization (Figure 2). The grasshopper studies estimated the influence of genetic versus non-genetic factors on the ocellar interneurons network by comparing various isogenic lines. These studies revealed consistent deviations in neuron count among isogenic grasshopper lines, highlighting the role of stochastic variation in the establishment of neural networks. While axonal pathways to target regions remained genetically determined and exhibited minimal variability, the spatial distribution of ocellar interneurons within the ganglia exhibited significant stochastic variability (Lai et al., 2022). Similarly, as discussed above, the left/right wiring asymmetry in the visual system of fruit flies is stochastic and non-heritable in nature. This asymmetry directly influences how well an individual fly orients towards a visual object (Linneweber et al., 2020). Despite extensive research on organisms with simpler nervous systems, the inter-individual variance in the mammalian brain—such as differences in neuron number and circuit organization—and its functional consequences remain elusive. As a result, the relative contributions of genetics, environment, and stochastic factors to this variance are still poorly understood.

Herein lies the potential of the armadillo to elucidate inter-individual variance in the mammalian brain and how SDV influences this variance. The application of techniques such as magnetic resonance imaging and electrophysiological recordings can enable the anatomical and functional mapping of the armadillo brain (Scholl et al., 2017; Moffitt et al., 2023). With a reference assembly of the armadillo genome available (Rhie et al., 2021), the growing toolbox of next-generation sequencing approaches can be applied to the study of armadillos at both the single-cell and tissue levels. Additionally, CRISPR/Cas genome editing allows for targeted manipulations of gene expression and neural circuitry in armadillos. Collectively, these methodological advancements open numerous possibilities for using armadillos to understand how individual differences in brain structure and function arise and the extent to which these differences are shaped by genetic, environmental, and stochastic factors.

## Concluding remarks

No single line of study and no single model organism can provide all the answers to complex questions regarding the brain. Here, we propose that the nine-banded armadillo emerges as an important model for dissecting the origins and expressions of individuality in mammalian brain development. The unique reproductive biology of armadillos provides a rare opportunity to study the role of stochastic developmental processes in shaping neural diversity and, by extension, distinct cognitive and behavioral traits. This line of inquiry will advance our understanding of brain variability and will provide empirical evidence on the extent to which individuality in the mammalian brain stems from random, uncontrollable biological events. Through the study of the armadillo, we will gain a mechanistic appreciation for how stochastic processes underlie variability and individuality, helping us understand the essence of what makes each of us unique.

## Figures and Tables

**TABLE 1:** Terminology used in this article to refer to sources of phenotypic variation.

Source of variation	Explanation
Genetic variation	Variation that originates in changes in DNA sequence
Environmental variation	Variation that originates from the external environment
Stochastic developmental variation (SDV)	Residual variation that cannot be explained by genetic and environmental variation. Term coined by Gunter Vogt (2015). Other terminologies have been used to refer to this residual variation, including developmental noise, developmental variation, intangible variation, third component, and random noise.

## Data Availability

The dataset presented in this study can be found in online repositories or upon request. The names of the repository/repositories and accession number(s) can be found below: https://macbraingallery.yale.edu/collection6/, B66 (for rhesus monkey brain).

## BOX 1 Twin and adoption studies

In twin studies, the idea is to contrast trait resemblance of monozygotic twins, who share identical genetic makeup, with dizygotic twins, who are genetically as similar as regular siblings. The premise is that if genetics significantly influence a trait, monozygotic twins should exhibit a higher resemblance to each other compared to dizygotic twins. On the other hand, if shared environmental factors play a more substantial role, then both types of twins, when raised in the same environment, should display similar levels of resemblance. This methodology allows for an estimation of the relative contributions of genetics and shared environment to various traits, including those related to the brain, behavior, and psychological tendencies. Complementing twin studies, adoption studies can also estimate the effects of genetics and environment on human traits. For instance, by focusing on individuals who have been adopted and thus raised apart from their biological parents, adoption studies can isolate the influence of the environment from that of genetics. This is achieved by comparing adopted children to their biological families, with whom they share genes but not environments, and to their adoptive families, with whom they share environments but not genes.
